# The Characteristics and Functions of SSRs and SNPs Based on the Transcriptome of *Tuta absoluta* Exposed to Different Concentrations of Abamectin and Chlorantraniliprole

**DOI:** 10.3390/insects16050446

**Published:** 2025-04-24

**Authors:** Fanxue Zhang, Meimei Mu, Zonglin Wang, Haoran Zhang, Yilan Song, Rong Xiao

**Affiliations:** Guizhou Provincial Key Laboratory for Agricultural Pest Management of the Mountainous Region, Institute of Entomology, Guizhou University, Guiyang 550025, China; zhangfx1115@163.com (F.Z.); mxx1369@163.com (M.M.); wzl0lzw@163.com (Z.W.); king_zhr@163.com (H.Z.); songyilan1214@163.com (Y.S.)

**Keywords:** *Tuta absoluta*, transcriptome analysis, molecular marker, SSR, SNP

## Abstract

*Tuta absoluta* (Meyrick) is an important invasive pest that seriously affects the yield and quality of tomatoes. In this study, based on the previously obtained transcriptome data, a total of 25,123 SSR loci and 332,537 SNP loci were identified. The identified SSRs had occurrence and appearance frequencies of 28.62% and 40.60%, respectively. SSRs with a length equal to or greater than 12 bp constituted 58.25% of the total SSR loci. Through the analysis of GO, COG annotations, and the KEGG pathway database, it was revealed that the majority of SSR-transcripts were involved in fundamental cellular metabolic functions. In addition, the frequency of SNP occurrence was approximately one SNP locus per 175 base pairs (bp), and the transition type was the main variant type. The functional annotations of SNP-transcripts were primarily concentrated in biological synthesis pathways such as peroxisome, RNA transport, carbon metabolism, and protein processing in the endoplasmic reticulum. These synthesis pathways are involved in the detoxification mechanism of *T. absoluta* and contribute to its enhanced survival under pesticide susceptibility. These findings provide valuable data for constructing genetic maps, assessing genetic diversity, and determining functional orientation in insects, and they also provide basic data for the molecular mechanism of *T. absoluta* ‘s response to pesticide susceptibility.

## Simple Summary

*Tuta absoluta* (Meyrick) is a globally distributed invasive pest. Its larvae form tunnels in leaves when feeding on the leaf mesophyll. In the research based on full-length transcriptome and comparative transcriptome data for *T. absoluta* by using the combined sequencing technology, a total of 25,123 SSR loci and 332,537 SNP loci were identified. Through the analysis of GO, COG annotations, and the KEGG pathway database, it was revealed that the majority of SSR-transcripts and SNP-transcripts were involved in fundamental cellular metabolic functions. These findings might help us understand how *T. absoluta* adapts to or resists abamectin and chlorantraniliprole.

## 1. Introduction

*Tuta absoluta* (Meyrick) (Lepidoptera: Gelechiidae) is an invasive pest that poses a serious threat to the tomato industry, significantly reducing the yield and economic benefits of tomatoes. Originating in South America [1], *T. absoluta* was first detected in Xinjiang, China, in 2017 and has since spread to Yunnan, Gansu, and other provinces in China [2,3]. *T. absoluta* mainly bores into leaves, leaving only the upper and lower epidermis of the leaves [4]. *T. absoluta* not only targets tomatoes but also attacks Solanaceae plants such as potatoes, eggplants, and tobacco [5,6,7]. It is difficult to control *T. absoluta* under suitable environmental conditions [8,9]. At present, the control of *T. absoluta* mainly relies on insecticides. A variety of pesticides, such as abamectin, chlorantraniliprole, and tetraniliprole, all have control effects on *T. absoluta* [10,11,12]. However, the frequent use of a single insecticide has led to a rapid increase in its resistance. The study found that *T. absoluta* has a certain degree of resistance to chlorantraniliprole (CAP) [13], indoxacarb [14], flubendiamide, and cyantraniliprole [15]. In addition to the tolerance of its gut symbionts to pesticides [13], the development of insecticide resistance in *T. absoluta* is also related to changes in the sensitivity of target sites, the enhancement of its detoxification ability, and simple genetic variation [16]. Compared to using a single active ingredient, using several active ingredients with different modes of insecticidal action may help delay the development of resistance. The use of compound pesticides is an important means to delay the development of its resistance. Some studies have found that, when the biopesticides Dipel, XenTari, and Agree are combined with lambda-cyhalothrin, they had a higher potential than the original formulations alone, with an approximately 3.67–10.08-fold impact on larval mortality [17]. The combination of abamectin and chlorantraniliprole can significantly enhance insecticidal activity and delay the increase in drug resistance [18]. However, the development of insecticide resistance in pests can only be postponed rather than eradicated, and the same is true for compound pesticides.

Microsatellites, simple sequence repeats (SSRs), are common and widespread DNA elements in the genomes of many organisms [19]. SSRs possess numerous advantages, such as high polymorphism, heterozygosity, wide distribution, rich variation, codominant inheritance, and rapid, convenient detection. Consequently, SSRs have been widely used in genetic linkage construction, gene mapping, fingerprint analysis, and diversity evaluations [20,21]. Some studies have found that large numbers of SSRs are in synteny and thus could be exploited as a tool to investigate genome structure and evolution [14]. SSR loci of various insects have been successfully mined based on transcriptome data. For instance, Prajapati et al. searched for and found 1913 potential SSRs from 30,451 transcripts of *Hellula undalis* (Lepidoptera: Pyralidae). Among these, 129 SSRs were classified as having a compound structure [22]. They considered that these sequences could be used as genetic markers for conducting further research on genetic improvement. Kattupalli et al. conducted a genome analysis of *Scirpophaga incertulas* (Lepidoptera: Pyralidae) and identified 21,696 SSR loci suitable for analyzing population diversity in rice-cultivation regions [23]. Miao et al. used 518 SSR markers to construct a genetic linkage map for *Bombyx mori* (Lepidoptera: Bombycidae) [24]. Huo et al. reported a new integrated strategy named the accurate microsatellite genotyping tool based on targeted sequencing (AMGT-TS) and provided a user-friendly web-based platform and a command-line version of AMGT-TS [25]. This method is not only useful for the background evaluation of genetic resources but also expands our understanding of the unintended effects of different genetic engineering techniques. Moghaieb et al. identified genotype-specific SSR markers that can be applied to enhance the characteristics related to stem borers in rice [26]. The information obtained provides a way for biologists to design novel pest management strategies as well as for the industry to design new classes of safer and specific insecticide molecules. In general, the successfully mined SSR loci are helpful for understanding the population genetic structure and migration patterns of insects, and they are crucial for deepening our understanding of their evolutionary processes.

A single nucleotide polymorphism (SNP) refers to the polymorphism of a DNA sequence caused by single nucleotide variation, namely, base-substitution mutations, including transitions, transversions, insertions, and deletions, which result in nucleic-acid sequence polymorphisms. It is a common and useful type of molecular genetic marker. SNPs account for more than 90% of all known polymorphisms [27]. Their higher availability and stability compared to SSRs provide enhanced possibilities for genetic and breeding applications, such as cultivar identification, construction of genetic maps, the assessment of genetic diversity, the detection of genotype/phenotype associations, and marker-assisted breeding [28]. SNP loci of various insects have been successfully mined based on transcriptome data. For instance, Zhu et al. identified 15,496 SNPs from the transcriptomes of deltamethrin resistant and susceptible *Anopheles sinensis* (Diptera: Culicidae) mosquitoes [29]. The identified SNP markers provide useful tools for future population genetic and comparative genomic analyses of malaria vectors. Xu et al. investigated the transcriptional profiles of deltamethrin-resistant and susceptible *Aedes albopictus* (Diptera: Culicidae) by performing paired-end sequencing for RNA expression analysis [30]. These findings laid a useful foundation for further studies on insecticide resistance mechanisms. SNPs can be used to assist in gene mapping and cloning to identify genes associated with important traits in insects, such as insecticide resistance, development, and reproduction. They can also be applied in molecular marker-assisted selection to accelerate the breeding process. Kassa et al. developed SNP markers for the *Sm1* gene resistant to orange wheat blossom midge (OWBM). These SNPs are a major improvement for the marker-assisted selection (MAS) of *Sm1* in wheat-breeding programs [31]. Chang and Hartman identified SNP polymorphic markers for the Mexican Bean Beetle (MBB), Potato Leafhopper (PLH), Soybean Looper (SBL), and Velvetbean Caterpillar (VBC), and they highlighted several leucine-rich repeat-containing genes and myeloblastosis transcription factors within the high linkage disequilibrium region surrounding significant SNP markers [32]. This information contributes to the research on the positioning of resistance to soybean pests. Some studies have found that several SNPs related to insecticide resistance have been identified in *Aphis gossypii* [33], *Myzus persicae*, and *Musca domestica* [34].

SSRs possess characteristics like high polymorphism and have been extensively used in genetic research. Some studies have revealed that SSRs can function as a tool for exploring genome structure and evolution. Through the mining of SSR loci, it becomes possible to understand the population genetic structure and migration patterns of insects and, thereby, indirectly gain an understanding of the development of their resistance. This is due to the fact that alterations in population structure might be associated with the evolution of resistance. SNPs display nucleic acid polymorphism. In certain studies, SNPs have been identified from the transcriptomes of insects presenting resistance or susceptibility to insecticides, such as *A*. *sinensis* and *A*. *albopictus*. This suggests that SNPs can be employed to aid in the identification of genes related to important traits in insects, for example, insecticide resistance. The resistance mechanism of insects to insecticides can be analyzed by detecting SNPs.

Some researchers have analyzed the comprehensive sex- and tissue-specific transcriptome of adult *T. absoluta*, the proteome of *T. absoluta* larvae infected with entomopathogenic fungi, the whole genome of odorant receptors in *T. absoluta*, etc. Lewald et al. also conducted a genome-wide population analysis of *T. absoluta* samples collected from various locations in Latin America [35,36,37,38]. However, to our knowledge, there has been no report on the analysis of the characteristics and functions of the SSRs and SNPs of *T. absoluta*. In this study, SSRs and SNPs were searched for in the transcriptome data of *T. absoluta* under pesticide susceptibility, and the gene functions were annotated. This study aims to lay a theoretical foundation for understanding the genetic characteristics of *T. absoluta*, developing SSR and SNP molecular markers and exploring the population genetic structure of *T. absoluta*. It also provides basic data for further exploring the molecular mechanism of the response of *T. absoluta* to insecticide mixtures.

## 2. Materials and Methods

### 2.1. Insects

The *T. absoluta* adults were collected in Baiyan Town, Puding County, Anshun City, Guizhou Province, China in September 2021. After collection, they were brought to the laboratory climate room at the Institute of Entomology of Guizhou University. In the laboratory, the adults were raised under certain conditions, the temperature was maintained at 25 ± 1 °C, with a relative humidity of 60 ± 5%, and the photoperiod followed a 16: 8 (L: D) hour cycle. For larval feeding, the cultivated tomato plants were placed in the climate chamber. To provide supplementary nutrition, the adults were given honey water, with a ratio of 9 parts water to 1 part honey. The tomato plants used for the experiment were cultured in the climate chamber of the Institute of Entomology, Guizhou University (the temperature was maintained at 25 ± 1 °C, with a relative humidity of 60 ± 5%, and the photoperiod followed a 16: 8 (L: D) hour cycle).

### 2.2. Sample Processing and RNA Isolation

In a previous study, we measured the sublethal concentrations LC_10_ (3.47 mgL^−1^) and LC_30_ (11.86 mgL^−1^) and the median lethal concentration LC_50_ (27.791 mgL^−1^) of abamectin and chlorantraniliprole on *T. absoluta* using toxicity regression equations [18]. The data we used in our current study were sourced from the work by Liu et al. (2023) [18]. The insecticide solutions were diluted in water containing 0.01% Tween-80 (Tianjin Kemiou Chemical Reagent, Tianjin, China), distilled water, or 0.01% Tween-80 without insecticides as a control. The insecticide was applied via the leaf-dipping method, and a piece of tomato leaf (we selected leaves that were fully expanded and showed no obvious signs of disease, pest damage, or nutrient deficiency from healthy and mature tomato plants) was immersed in different concentrations for 15 s. After the leaf was dried, it was placed in a 12 cm sterile petri dish, and 40 third-instar larvae were added. The petri dish was sealed with plastic wrap, and holes were made in it for ventilation. After 24 h of treatment, 15 surviving larvae were randomly selected as a biological replicate, and each group consisted of three biological replicates. The collected samples were frozen in liquid nitrogen and stored at −80 °C in a refrigerator. Total RNA was isolated using TRIGene Reagent (Genstar, Beijing, China). A 1% agarose gel was used to detect RNA integrity and contamination. The concentration and quality were measured using a Nanodrop 2000 spectrophotometer (Thermo Fisher Scientific Inc., Waltham, MA, USA).

### 2.3. Sequencing and Data Analysis

In a previous study, we obtained transcriptome data from third-instar larvae of *T. absoluta* exposed to various concentrations (LC_10_, LC_30_, LC_50_, CK) of abamectin and chlorantraniliprole using a combination of PacBio Iso-Seq and Illumina RNA-seq technologies [18].

PacBio Iso-Seq and Illumina RNA-Seq were both employed in this study due to their complementary advantages. PacBio Iso-Seq is well-known for its capacity to generate long-read sequences, which is vital for acquiring full-length transcripts. This enables a more thorough comprehension of the gene structures associated with SSRs. Conversely, Illumina RNA-Seq provides high-throughput and high-accuracy short-read sequencing. It is highly efficient in quantifying gene expression levels, which is indispensable for analyzing the differential expression patterns linked to SNPs under diverse pesticide susceptibility conditions. By integrating these two techniques, we were not only able to accurately identify and characterize the SSR-containing genes but also comprehensively analyze the transcriptional alterations related to SNPs in *T. absoluta*. This offers a more profound and accurate perspective on the molecular mechanisms underlying the insect’s response to pesticides.

For PacBio Iso-Seq sequencing, equal amounts of total RNA from each treatment (CK, LC_10_, LC_30_, and LC_50_) were pooled and then sequenced. Transcriptome sequences with a length greater than 500 bp were screened for SSR analysis using MISA software (http://pgrc.ipk-gatersleben.de/misa/). Transcriptome sequences were analyzed for repeat motifs ranging from mononucleotide to hexanucleotide. Screening parameters were established as follows: mononucleotide sequences were replicated at least 10 times, dinucleotide sequences at least 6 times, and trinucleotide, tetranucleotide, pentanucleotide, and hexanucleotide sequences at least 5 times. If the distance between two SSRs was less than 100 bp, a compound SSR locus was considered to be formed.

For Illumina RNA sequencing, 12 libraries were prepared, with their RNA samples sourced from four different concentrations (CK, LC_10_, LC_30_, and LC_50_), and then sequencing was carried out. All qualified samples had their libraries constructed and were sequenced on the Illumina NovaSeq 6000 sequencing platform. Based on the transcriptome data, we used STAR software (https://github.com/alexdobin/STAR/) to align the reads of each sample with the transcript sequence. Then, GATK software (https://gatk.broadinstitute.org/hc/en-us) was used to identify SNP loci. The total length of transcripts containing SNPs (SNP-transcripts) was counted using seqkit software (https://bioinf.shenwei.me/seqkit/). The command was: $seqkit stats *.fa, and the coding region length of SNP-transcripts was counted in the same way to obtain the result. We used Microsoft Excel 2019 to analyze the number of SNP transitions and transversions in the SNP-transcripts (the intersection of biological repeat groups was used as the statistical value).

Based on the obtained transcriptome, transcripts containing SSR-transcripts and SNP-transcripts were aligned with the Gene Ontology (GO) [39], Clusters of Orthologous Groups (COG) [40], and Kyoto Encyclopedia of Genes and Genomes (KEGG) databases using DIAMOND software (https://github.com/bbuchfink/diamond) to annotate the transcripts [41]. We used Blast software (https://blast.ncbi.nlm.nih.gov/) to compare the transcripts with the databases (Blast parameters: E-value not greater than 10^−5^) to obtain annotation information for each SSR-transcript or SNP-transcript.

The transcriptome sequence data from *T. absoluta* are available in the NCBI SRA database (project number PRJNA869533).

## 3. Results

### 3.1. SSR Distribution of Transcriptome

By screening transcripts longer than 500 bp, a total of 60,386 transcripts were included in the analysis, and 25,123 SSRs were identified, of which 2028 were compound forms. In the *T. absoluta* transcriptome, an SSR locus appeared on average every 6547 bp. The total number of high-quality (HQ) transcripts containing SSRs was 17,284, of which 4824 transcripts contained more than one SSR locus (Table 1). The frequency of SSR occurrence was 28.62%, calculated as the total number of high-quality transcriptomes containing SSRs divided by the total number of high-quality transcripts detected, multiplied by 100%. The frequency of SSR occurrence was 41.60%, determined by dividing the total number of SSRs identified by the total number of high-quality transcriptomes detected and then multiplying by 100%.

### 3.2. Analysis of Different Repeat Types of SSR Loci

The classification of SSR loci repeat types in the *T. absoluta* transcriptome was carried out. Six nucleotide types were identified. The number of SSR loci formed by different repeat types showed significant variation (Table 2 and Table S1). Among them, the number of mononucleotide repeat SSRs (13,810) was the highest, accounting for 54.97% of the total SSR loci, and the most frequent repeat unit ‘A’ numbered 6670, representing 48.30% of the mononucleotide repeat loci. The number of dinucleotide repeat SSRs was 4475, which accounted for 17.81% of the total SSR loci, and the most abundant repeat units were ‘CGC’, with a count of 331, accounting for 7.25% of the trinucleotide repeat loci. The number of trinucleotide repeat SSRs was 4567, accounting for 18.18% of the total SSR loci, and the most abundant repeat units ‘CGC’ were 331, accounting for 7.25% of the trinucleotide repeat loci. The number of tetranucleotide repeat SSRs was 1944, accounting for 7.74% of the total SSR loci, and the repeat units ‘TATG’ numbered 356, representing 18.31% of the tetranucleotide repeat loci. The numbers of pentanucleotide and hexanucleotide repeat SSRs were the lowest, accounting for 0.71% and 0.59% of the total SSR loci, respectively.

The frequencies of SSRs formed by different repeat types varied significantly, with mononucleotide repeats showing the highest frequency. On average, there were 22.87 SSR loci per 100 transcripts. SSRs with hexanucleotide repeats had the lowest frequency, averaging just 0.25 loci per 100 transcripts (Table 2).

### 3.3. Analysis of SSR Motif Types and Repeat Times

A total of 110 SSR repeat motifs were identified, including 2 mononucleotides, 4 dinucleotides, 10 trinucleotides, 24 tetranucleotides, 36 pentanucleotides, and 34 hexanucleotides. Furthermore, the number of SSR loci formed by different motif types varied significantly. Generally, the number of SSR loci decreased as the number of nucleobases increased within the motifs. Specifically, the mononucleotide motif (A/T) had the highest frequency at 21.37%, while the lowest frequency was a mere 0.0017% for tetranucleotide, pentanucleotide, and hexanucleotide motifs (Table S2).

**Figure 1:**
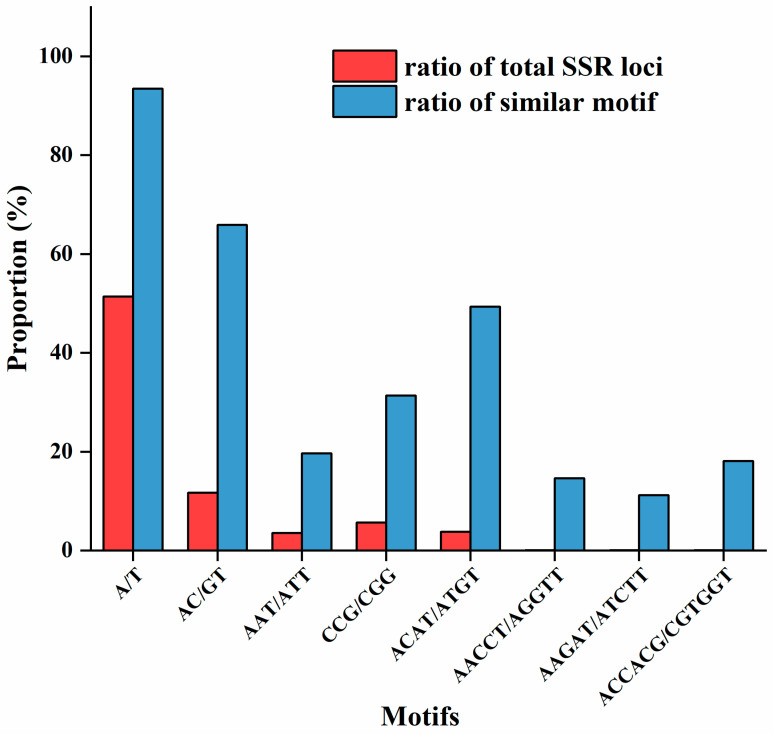
The distribution of the number of SSR loci in the dominant motifs of different repeat types in *T. absoluta*.

The dominant A/T motif in mononucleotides numbered 12,907, constituting 51.38% of the total SSR loci and 93.46% of mononucleotide SSRs. The dominant AC/GT motif in dinucleotides numbered 2949, representing 11.74% of the total SSR loci and 65.90% of dinucleotide SSRs. The number of SSR loci with the major CCG/CGG motif in trinucleotides was 1432, accounting for 5.70% of the total SSR loci and 31.36% of trinucleotide SSRs. The dominant ACAT/ATGT motif in tetranucleotides numbered 960, constituting 3.82% of the total SSR loci and 49.38% of tetranucleotide SSRs. Among pentanucleotide repeat motifs, the dominant ones were AACCT/AGGTT and AAGAT/ATCTT, numbering 26 and 20, and accounting for 0.103% and 0.08% of the total SSR loci, respectively. The number of SSR loci with the dominant ACCACG/CGTGGT motif in hexanucleotides was 27, contributing 0.107% to the total SSR loci (Table S2, Figure 1).

SSR motif repeat counts varied among different types, leading to diverse formations of SSR loci. The variation in SSR repeat counts influenced SSR polymorphism by altering the length of SSRs. SSR motifs typically repeated from 6 to 12 times (Table S2). Mononucleotide motifs most commonly repeated from 10 to 12 times, with 11,699 SSR loci identified within this interval, comprising 46.57% of the total SSR loci. Dinucleotide motifs most frequently repeated between 6 and 12 times, encompassing 3722 SSR loci, which amounted to 14.82% of the total SSR loci. Trinucleotide motifs predominantly repeated between 5 and 9 times, including 4488 SSR loci, and these loci accounted for 17.86% of the total SSR loci. Tetranucleotide motifs typically repeated between 6 and 12 times, with a total of 1900 SSR loci falling within this range, representing 7.8% of the total SSR loci. Repeat counts for pentanucleotide and hexanucleotide motifs were widely distributed, with the repeat counts of 327 SSRs ranging from 6 to 55.

### 3.4. Distribution Characteristics of SSR Motif Length

**Figure 2:**
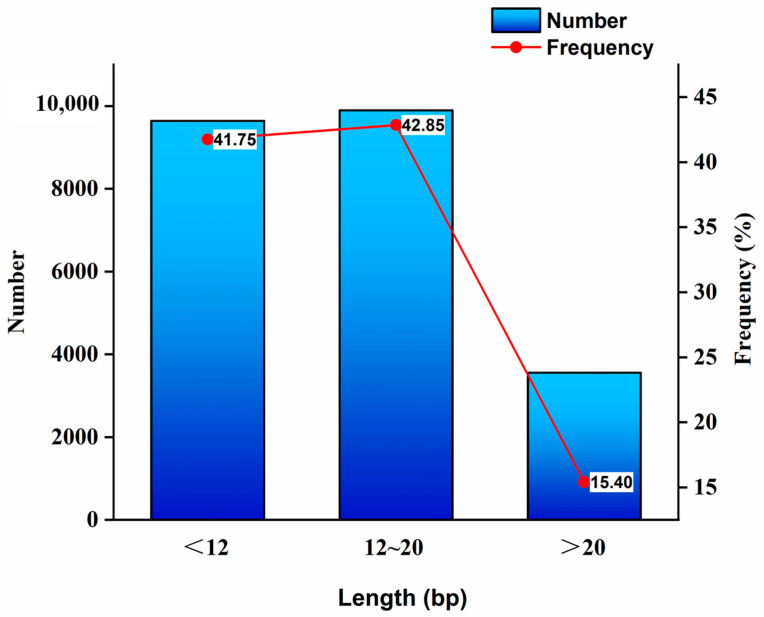
Length distribution of microsatellites of SSR in *T. absoluta*.

The length of the SSR motif exhibited a positive correlation with polymorphism. Analysis of the *T. absoluta* transcriptome data revealed that the SSR motif lengths were primarily distributed within the range of 10–20 bp (Figure 2). SSRs with lengths of less than 12 bp numbered 9642, representing 41.75% of the total SSR loci. SSRs with lengths ranging from 12 to 20 bp totaled 9897, accounting for 42.85% of the total SSR loci. SSRs with lengths exceeding 20 bp were 3556 in number, constituting 15.40% of the total SSR loci.

### 3.5. GO Functional Classification of of SSR-Transcripts

A total of 17,284 transcripts with SSR-transcripts were annotated in the GO database, with 8916 (51.59%) successful annotations. The GO database categorizes gene functions into cellular components, molecular functions, and biological processes. Further classification and analysis of gene function indicated that the cellular component category comprised 16 subcategories, with ‘cell’ and ‘cell part’ encompassing 2777 SSR-transcripts, accounting for 31.15% of annotated transcripts in GO, followed by ‘organelle’ with 2118 annotations (23.76%). The smallest category was ‘cell junctions’ with 36 annotations. Molecular function encompassed 13 subcategories, with ‘binding’ having the highest number of annotations at 4855 (54.45%), followed by ‘catalytic activity’ with 3806 (42.69%), and ‘morphogen activity’ with the fewest at only 2. The biological process category included 21 subcategories, with ‘cellular process’ having the most annotations at 4028 (45.18%), followed by ‘metabolic process’ with 3597 (40.34%), and ‘rhythmic process’ with the fewest at 11 (Figure S1).

### 3.6. COG Pathway Analysis of SSR-Transcripts

The SSR-transcripts were compared with the COG database for annotation and classification. The results indicated that, out of 17,284 SSR-transcripts, 2555 were successfully annotated. Based on functional classification, these could be divided into 23 categories. The analysis revealed that most SSR-transcripts were implicated in cellular life activities, with substantial involvement in functional categories such as O: Posttranslational modification, protein turnover, chaperones; J: Translation, ribosomal structure, and biogenesis; and G: Carbohydrate transport and metabolism, which accounted for 13.58% (347), 10.92% (279), and 10.10% (258) of the SSR-transcripts, respectively. The functional categories with less representation included U: Intracellular trafficking, secretion, and vesicular transport; Z: Cytoskeleton; and A: RNA processing and modification, which accounted for only 0.27%, 0.20%, and 0.08% of SSR-transcripts, respectively (Figure S2).

### 3.7. KEGG Metabolic Pathway Analysis of SSR-Transcripts

To fully comprehend the functional and pathway enrichment of the transcripts found in *T. absoluta*, 17,284 SSR-transcripts were annotated in the KEGG database. Among them, 4894 (28.32%) were successfully annotated across the following six functional categories: metabolism, organismal systems, genetic information processing, human diseases, cellular processes, and environmental information processing. Among these categories, the number of sequences in the metabolism category was the largest, accounting for 10.66% (1842) of the SSR-transcripts, followed by genetic information processing, accounting for 7.30% (1261). The number of sequences in the human diseases category was the smallest, accounting for 0.44% (76) (Table S3).

A more detailed classification analysis of gene function was conducted. Metabolism featured 90 secondary categorization processes, and there was a significant variation in the number of annotations. Secondary classification results revealed that SSR-transcripts related to lysine degradation and fatty acid metabolism had the highest number of annotations, constituting 4.05% and 3.78% of KEGG-annotated SSR-transcripts, respectively. The fewest annotations were identified in penicillin and cephalosporin biosynthesis, glycosphingolipid biosynthesis-ganglio series, D-arginine and D-ornithine metabolism, and biotin metabolism, with only one SSR-transcript annotated. Organismal systems encompassed 38 secondary classifications, with the Toll and Imd signaling pathways containing the most SSR-transcripts, accounting for 1.88% of KEGG-annotated SSR-transcripts. Processing included 22 secondary classifications, with RNA transport and protein processing in the endoplasmic reticulum having the greatest number of SSR-transcripts, comprising 5.56% (272) and 5.46% (267) of KEGG-annotated SSR-transcripts, respectively. Human diseases featured 20 secondary classifications, with salmonella infection having the most annotations, while the minimum number was only one. Cellular processes included 16 secondary classifications, with autophagy in animals having the most annotations, representing 5.13% (251) of KEGG-annotated SSR-transcripts. Environmental information processing was divided into 26 secondary classifications, with the MAPK signaling pathway-fly having the largest number of annotations, constituting 3.46% (173) of the KEGG-annotated SSR-transcripts (Figure S3).

### 3.8. SNP Loci Analysis

**Figure 3:**
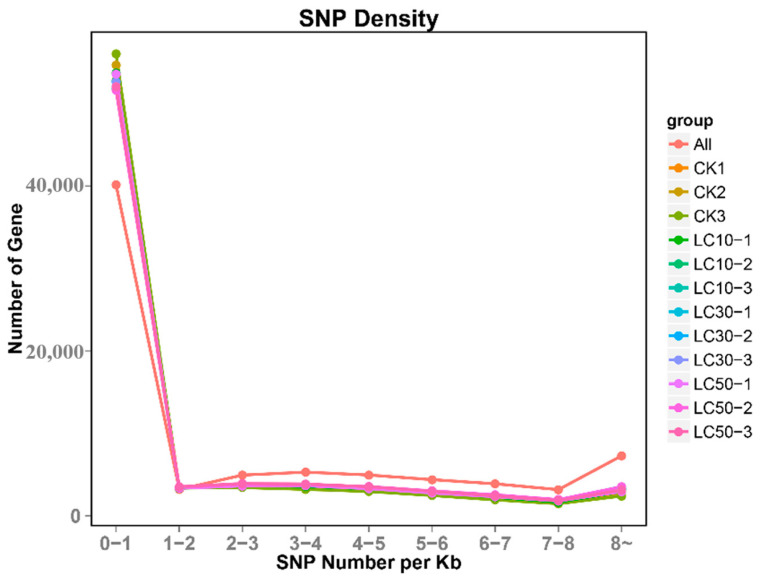
Density frequency distribution of SNPs.

The analysis of SNP loci showed that there was a total of 332,537 SNP loci in the 12 transcriptome libraries. Among these, there were 189,654 SNP loci in various genotypes of the control group. In the LC_10_ treatment group, there were 214,441 SNP loci; in the LC_30_ treatment group, there were 211,371 SNP loci; and in the LC_50_ treatment group, there were 213,478 SNP loci. Among the 77,216 unigenes, 37,626 were SNP-transcripts. Based on the number of alleles at the SNP locus and the number of different bases supported by the sequencing reads, SNP loci could be divided into homozygous SNP loci (only one allele) and heterozygous SNP loci (two or more alleles). According to the situation of SNP-transcripts in each treatment group, the total length of SNP-transcripts was 58,261,243 bp, the coding region length was 26,500,542 bp, and the non-coding region length accounted for 31,760,701 bp. The SNP occurrence frequency was approximately 1 per 175 bp (that is, approximately one SNP locus per 175 bp) (Table S4). Frequency analysis of SNP density showed that unigenes with zero to two SNP loci per kilobase pair had the highest frequency (Figure 3).

### 3.9. SNP Transitions and Transversions

**Figure 4:**
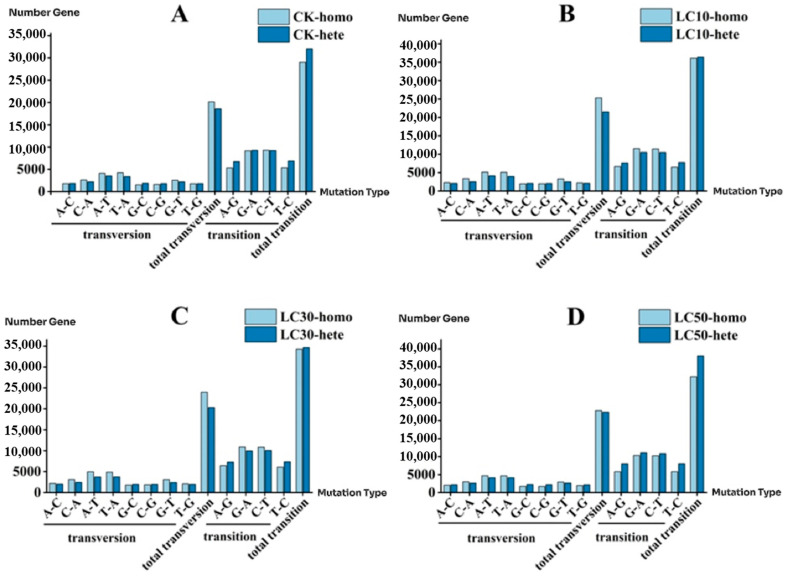
Numbers of different SNP mutation types ((**A**):the number of SNP mutation types under the CK; (**B**): the number of SNP mutation types under the LC_10_; (**C**): the number of SNP mutation types under the LC_30_; (**D**): the number of SNP mutation types under the LC_50_) (CK-homo: CK-homozygous; CK-hete: CK-heterozygous; LC_10_-homo: LC_10_-homozygous; LC_10_-hete: LC_10_-heterozygous; LC_30_-homo: LC_30_-homozygous: LC_30_-hete: LC_30_-heterozygous; LC_50_-homo: LC_50_-homozygous: LC_50_-hete: LC_50_-heterozygous).

The quantitative analysis of the mutation types of *T. absoluta* SNPs showed that the number of transitions was higher than that of transversions in each treatment group. In homozygous SNP loci, the CK treatment group had 29,051 transition types (transition frequency = 59.03%) and 20,162 transversion types (transversion frequency = 40.97%) (Figure 4A). The LC_10_ treatment group had 36,083 transition types (transition frequency = 58.79%) and 25,292 transversion types (transversion frequency = 41.21%) (Figure 4B). The LC_30_ treatment group had 34,276 transition types (transition frequency = 58.84%) and 23,976 transversion types (transversion frequency = 41.16%) (Figure 4C). The LC_50_ treatment group had 32,210 transition types (transition frequency = 58.59%) and 22,768 transversion types (transversion frequency = 41.41%) (Figure 4D). In heterozygous SNP loci, the CK treatment group had 32,041 transition types (transition frequency = 63.29%) and 18,588 transversion types (transversion frequency = 36.71%) (Figure 4A). The LC_10_ treatment group had 36,378 transition types (transition frequency = 62.86%) and 21,497 transversion types (transversion frequency = 37.14%) (Figure 4B). The LC_30_ treatment group had 34,666 transition types (transition frequency = 63.09%) and 20,285 transversion types (transversion frequency = 36.91%) (Figure 4C). The LC_50_ treatment group had 38,049 transition types (transition frequency = 62.99%) and 22,358 transversion types (transversion frequency = 37.01%) (Figure 4D).

### 3.10. GO Functional Classification of SNP-Transcripts

**Figure 5:**
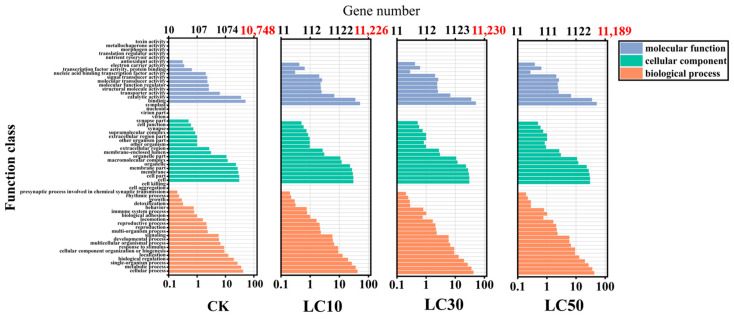
GO functional classification of SNP-transcripts in *T. absoluta*.

A total of 37,626 transcripts with SNP-transcripts were annotated in the GO database, with 13,974 (37.14%) successful annotations. The control group and each treatment group were analyzed in terms of the three main functional categories of GO. The types of the annotated items in each treatment group were the same, but the gene quantities might vary. Most abundant was the biological process (annotated to include 22 secondary classifications, accounting for 40.0%), followed by the cellular component (annotated to include 18 secondary classifications, accounting for 32.73%), and molecular function (annotated to include 15 secondary classifications, accounting for 27.27%). There were 10,748 SNP-transcripts annotated in the CK, with the most annotated function being binding with 5422 (50.45%), followed by cellular process with 4459 (41.49%), metabolic process with 3840 (35.73%), and catalytic activity with 3782 (35.19%). In the LC_10_ treatment group, there were 11,226 annotations, with the most annotated function being binding with 5668 (50.49%), followed by cellular process with 4674 (41.64%), metabolic process with 4022 (35.83%), and catalytic activity with 3933 (35.03%). In the LC_30_ treatment group, there were 11,230 annotations, with the most annotated function being binding with 5632 (50.15%), followed by cellular process with 4657 (41.47%), metabolic process with 3991 (35.54%), and catalytic activity with 3920 (34.91%). In the LC_50_ treatment group, there were 11,189 annotations, with the most annotated function being binding with 5673 (50.70%), followed by cellular process with 4672 (41.76%), metabolic process with 3993 (35.69%), and catalytic activity with 3911 (35.69%) (Figure 5). The results showed that, through the GO functional classification of the SNP-transcripts in the control group and each treatment group, the biological process pathway had the highest proportion of gene numbers, with the cellular process pathway having the most gene numbers in its secondary classification, followed by the cellular component pathway, with the cell and cell part pathways having the most numbers in its secondary classification, and the molecular function pathway, with the binding pathway having the most gene numbers. However, in terms of quantity proportion, the number of enriched genes in each GO item under the stress of the pesticide mixture treatment was greater than that of the CK (Figure S4).

### 3.11. COG Pathway Analysis of SNP-Transcripts

The sequences of transcripts containing SNP-transcripts were annotated and classified in the COG gene database. A total of 3016, 3058, 3077, and 3037 SNP-transcripts were annotated in CK, LC_10_, LC_30_, and LC_50_, respectively, and most of the annotated SNP-transcripts participated in two or more functions. These SNP-transcripts were involved in most life activities or functions. The functions with greater participation were O: Posttranslational modification, protein turnover, and chaperones, which accounted for 13.46% (406) of the CK, 13.15% (402) of the LC_10_, 13.16% (405) of the LC_30_, and 13.01% (395) of the LC_50_, respectively; G: Carbohydrate transport and metabolism, which accounted for 11.41% (344) of the CK, 11.41% (349) of the LC_10_, 11.50% (354) of the LC_30_, and 11.33% (344) of the LC_50_, respectively; J: Translation, ribosomal structure, and biogenesis, which accounted for 10.78% (325) of the CK, 10.46% (320) of the LC_10_, 10.40% (320) of the LC_30_, and 10.60% (322) of the LC_50_, respectively (Figure S5). In the CK and each treatment group, the types of COG annotations and the order of gene enrichment quantities were the same, but the total number of annotated SNP-transcripts in the pesticide mixture treatment group was greater than that of the CK, and there was a difference in the amount of gene enrichment in specific items.

### 3.12. KEGG Metabolic Pathway Analysis of SNP-Transcripts

**Figure 6:**
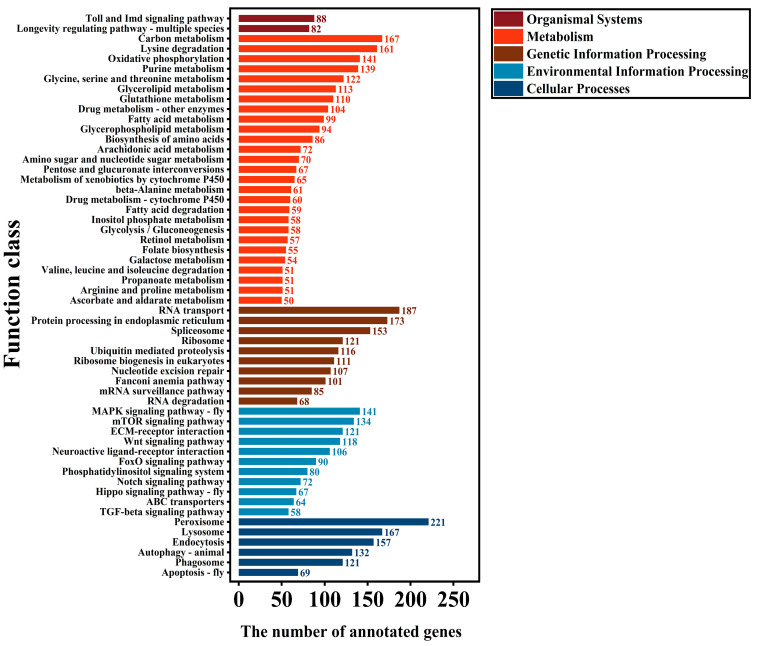
KEGG pathway analysis of SNP-transcripts for the LC_10_ treatment of *T. absoluta*.

The KEGG metabolic pathways of transcripts containing SNP-transcripts were annotated. The results showed that 8134 SNP-transcripts were successfully annotated. The annotation results were categorized into five major metabolic pathways, namely cellular processes, environmental information processing, genetic information processing, metabolism, and organismal systems. A total of 4587 SNP-transcripts participated in 269 known metabolic or signaling pathways in CK, of which the largest proportion was peroxisome, accounting for 4.51% (207), followed by RNA transport, accounting for 3.97% (182), and protein processing in the endoplasmic reticulum, accounting for 3.84% (176) (Figure S6). In the LC_10_ treatment group, a total of 4755 SNP-transcripts were annotated, participating in 262 known metabolic or signaling pathways, of which the largest proportions were peroxisome, accounting for 4.65% (221), followed by RNA transport, accounting for 3.93% (187), and protein processing in the endoplasmic reticulum, accounting for 3.64% (173) (Figure 6). In the LC_30_ treatment group, 4767 SNP-transcripts were annotated, participating in 264 known metabolic or signaling pathways, with the largest proportions being peroxisome, accounting for 4.47% (213), followed by RNA transport, accounting for 3.90% (186), and protein processing in the endoplasmic reticulum, accounting for 3.63% (173) (Figure S7). In the LC_50_ treatment group, a total of 4735 SNP-transcripts were annotated, participating in 260 known metabolic or signaling pathways, with the largest proportions being peroxisome, accounting for 4.60% (218), followed by RNA transport, accounting for 3.95% (187), and protein processing in the endoplasmic reticulum pathway, accounting for 3.63% (172) (Figure S8). All treatment groups of SNP-transcripts were significantly enriched in peroxisome, RNA transport, and protein processing in endoplasmic reticulum pathways, but the number of annotated genes in each pathway under pesticide treatment was more than that of the control group. The main pathways with increased numbers are peroxisome (LC_10_: 14, LC_30_: 6, LC_50_: 11), RNA transport (LC_10_: 5, LC_30_: 4, LC_50_: 5), oxidative phosphorylation (LC_10_: 24, LC_30_: 24, LC_50_: 14), and lysine degradation (LC_10_: 16, LC_30_: 8, LC_50_: 15). There were also individual pathways with opposite trends. Compared with the control treatment, the number of annotated genes in the protein processing in the endoplasmic reticulum pathway decreased by 3 (LC_10_, LC_30_) and 4 (LC_50_) in each pesticide treatment.

## 4. Discussion

Understanding the inheritance of *T. absoluta* is crucial for integrated management. In this study, by screening sequences longer than 500 bp, 25,123 SSRs were identified within 60,386 high-quality (HQ) transcripts, and these SSRs were distributed in 17,284 transcripts. The frequency of SSR occurrence was 28.62%, and the frequency of SSR appearance was 41.60%. These frequencies are higher than those reported for *Bactrocera dorsalis* (Diptera: Tephritidae) (occurrence: 13.96%, appearance: 16.12%) [42], *Rhyacionia leptotubula* (Lepidoptera: Tortricidae) (occurrence: 2.92%, appearance: 3.09%) [43], *Sogatella furcifera* (Hemiptera: Delphacidae) (occurrence: 7.26%, appearance: 8.96%) [44], and Sycamore Lace Bug *Corythucha ciliata* (Hemiptera: Tingidae) (occurrence: 19.88%, appearance: 28.68%) [45]. However, these frequencies are lower than those of *Holcocerus hippophaecolus* (Lepidoptera: Cossidae) (occurrence: 39.52%, appearance: 51.41%) [46]. The variation in these frequencies may be attributed to interspecies differences that influence the total gene count and the incidence of SSR occurrences. Additionally, the methodology of SSR locus search and the availability of databases also play a role [47].

By analyzing all the types of SSR motifs in *T. absoluta*, it was found that the mononucleotide motif types accounted for the largest proportion. Among them, the dominant A/T motif accounted for 51.38% of the total number of all SSR loci. There are many insects with SSR profiles similar to that of *T. absoluta*. For instance, the A/T motif represented 41.21% of the total SSRs in *Octodonta nipae* (Coleoptera: Hispidae) [48] and 28.57% in *Rhopalosiphum padi* (Hemiptera: Aphididae) [49]; however, this is in contrast to the most dominant motifs found in certain other insects. For example, CCG/CGG was the most dominant motif in *Mythimna separata* (Lepidoptera: Noctuidae) [50], AT/AT in *Riptortus pedestris* (Hemiptera: Coreidae) [51], and AT/TA in *Aphis aurantia* (Hemiptera: Aphididae) [52]. This discrepancy may arise from the search criteria for single-base repeats in *T. absoluta* transcripts, which required a minimum of ten repetitions. Additionally, the diversity and prevalence of DNA slippage mechanisms, mismatch repair systems, and transposable elements might contribute to the variation observed across different motif types [53].

The length of SSRs is positively correlated with polymorphism, and SSR length is the primary determinant of polymorphism [54]. SSRs with lengths less than 12 bp, between 12 and 20 bp, and more than 20 bp are indicative of low, moderate, and high polymorphism, respectively. In this study, SSRs were predominantly distributed within the range of 10 to 4537 bp, inclusive of mixed SSRs. This range exceeded that observed in *B. dorsalis* [43]. It is hypothesized that this extensive range is attributable to the presence of complex SSRs. SSRs with a length of less than 12 bp accounted for 41.75% of the total SSR loci, those between 12 and 20 bp accounted for 42.85%, and those exceeding 20 bp accounted for 15.40%, signifying that SSR loci with moderate polymorphism and high polymorphism accounted for 58.25% of the total SSR loci. This indicates that *T. absoluta* possesses numerous SSR loci with high polymorphism and significant development potential.

Analyzing the characteristics of SNPs in *T. absoluta*, a total of 332,537 SNP loci were identified on 37,626 SNP-transcripts in the transcriptome data of *T. absoluta*. The total length of the SNP-transcripts was 58,261,243 bp, and the coding region length was 26,500,542 bp. The non-coding region was 31,760,701 bp, and the SNP occurrence frequency was 0.00751 (1/175 bp). This mutation frequency was higher than that found in the human genome (1/1000 bp), *Vigna radiata* (L.) Wilczek (Fabales: Fabaceae) (1/860 bp) [55], wheat (1/540 bp) [56], and *Pardosa pseudoannulata* (Araneae: Lycosidae) (1/301 bp) [57], but lower than *Megalobrama amblycephala* (Cypriniformes: Cyprinidae) (1/158 bp) [58], *A. sinensis* (1/167 bp) [29], *Secale cereale* (Poales: Poaceae) (1/58 bp) [59], and *Vitis vinifera* L. (Vitales: Vitaceae) (1/64 bp) [28]. The main reasons for the difference in base mutation frequency are related to the species, genetic background, and species habitat.

In this study, the ratio of SNP transition types was approximately 60%, and the proportion of transversion types was approximately 40%. This result is similar to that of *Tetrastichus brontispae* (Hymenoptera: Eulophidae) (transition type 72.94%, transversion type 27.06%) [60], *Agrotis segetum* (Lepidoptera: Noctuidae) (transition type 64.02%, transversion type 35.98%) [61], the gonads of *Pelophylax nigromaculatus* (Anura: Ranidae) (transition type 63.63%, transversion type 36.37%) [62], and *Vigna mungo* (Fabales: Fabaceae) (transition type 61%, transversion type 39%) [63]. The reason for this result is that there is a preference for transition in the mutation types, which proves the evolutionary law that substitution is more likely to occur within the same type of base [64]. We found that some SNPs only existed in the abamectin–chlorantraniliprole mixture groups but not in the CK. In addition, after treatment with different concentrations of the abamectin–chlorantraniliprole mixture, the SNP loci of different treatment groups differed. This indicated that the mutations at the SNP loci of *T. absoluta* were subjected to different concentrations of abamectin–chlorantraniliprole, and the molecular mechanisms responding to different concentrations of the pesticide mixture were also different. Therefore, we analyzed the gene functions of responses to different concentrations of the pesticide mixture on SNP-transcripts.

Comparisons of SSR-transcripts and SNP-transcripts with the GO, COG, and KEGG databases revealed that SSR-transcripts in the GO database were predominantly involved in cellular binding, catalytic activity, and metabolic processes (Figure S1), while SNP-transcripts were predominantly involved in cellular binding, catalytic activity, and metabolic processes (Figure 5). According to COG annotations, SSR-transcripts and SNP-transcripts were principally involved in post-translational modification, protein turnover, chaperones, translation, ribosomal structure, biogenesis, as well as carbohydrate transport and metabolism (Figures S2 and S5). KEGG annotations indicated that most SSR-transcripts were associated with metabolism, genetic information processing, and cellular processes (Figure S3), and most SNP-transcripts were involved in multiple pathways such as peroxisome, RNA transport, carbon metabolism, and protein processing in the endoplasmic reticulum (Figure 6 and Figures S6–S8). The analysis of the GO, COG, and KEGG databases suggests that SSR-transcripts are primarily involved in the fundamental metabolic processes of cells, potentially helping *T. absoluta* in adapting to or resisting the abamectin and chlorantraniliprole (Figures S1–S3).

The insecticide resistance of *T. absoluta* mainly stems from changes in target locus sensitivity and enhanced detoxification capabilities. The specific augmentation in resistance depends on the mode of action of the active ingredient [16]. Some studies have demonstrated that the mutations *G4903E* and *I4746M* at the target loci of the ryanodine receptor (RyR) in *T. absoluta* have altered the affinity of the RyR for diamide insecticides, thereby resulting in an increase in its insecticide resistance [65]. Moreover, it has also been discovered that the development of resistance in *T. absoluta* is associated with the significant overexpression of a gene encoding uridine diphosphate glycosyltransferase (UGT) [66]. This study revealed that SSR-transcripts and SNP-transcripts are closely related to energy metabolism molecular mechanisms. The findings indicated that when *T. absoluta* is subjected to pesticide susceptibility, these SNP-transcripts participate in its various life activities, affecting its various biological traits, and they are involved in its pesticide detoxification mechanism (Figure 5, Figure 6 and Figures S5–S8).

In conclusion, the distribution frequencies of SSR and SNP loci in the transcriptome data of *T. absoluta* are relatively high. More than half of the SSRs exhibit moderate to high polymorphism, indicating that they could play a crucial role in the genome of the tomato leafminer. Analyses of GO, COG annotations, and KEGG pathways demonstrate that most SSR-transcripts are involved in basic cellular metabolic functions. This implies that SSRs might play an important role in the growth, development, and environmental adaptation of the tomato leafminer by influencing these fundamental metabolic processes. When compared with relevant studies on other insects, the frequency and distribution of SSRs in the tomato leafminer have certain specificity, which might be related to its unique biological characteristics and evolutionary history. The results of this study provide valuable data for constructing the genetic map of *T. absoluta*, evaluating genetic diversity, and determining functional localization. The functional annotation of SNP transcripts mainly concentrates on biosynthetic pathways such as peroxisome, RNA transport, carbon metabolism, and protein processing in the endoplasmic reticulum. These pathways are engaged in the detoxification mechanism of the tomato leafminer. This result not only reveals the molecular basis of *T. absoluta*’s response to pesticide susceptibility but also provides a theoretical basis for the development of molecular detection methods based on SNP markers. This study has clarified some key metabolic processes and detoxification mechanisms of *T. absoluta* in response to pesticide susceptibility. In subsequent research, new control strategies can be devised with a focus on these mechanisms, providing new targets for novel pesticides, which are less likely to generate cross-resistance with abamectin and chlorantraniliprole. Meanwhile, the development of molecular detection methods based on SNP markers enables the rapid and accurate detection of whether there are SNP loci associated with insecticide resistance in field populations of *T. absoluta*. This provides guidance for rational pesticide application, avoiding ineffective pesticide use and the further exacerbation of insecticide resistance.

## 5. Conclusions

In this study, by combining Illumina RNA-seq and PacBio Iso-Seq, we obtained the transcriptome data of the third-instar larvae of *T. absoluta* exposed to different concentrations of abamectin and chlorantraniliprole. SSR loci and SNP loci were identified, and the functions of the SSR-transcripts and SNP-transcripts were annotated.

The analysis revealed that SSR loci in *T. absoluta* are characterized by high occurrence frequency, a diversity of repeat types, a broad distribution of repeat numbers, and high polymorphism, and it indicated that the majority of SSR-transcripts are implicated in the fundamental metabolic functions of cells. These results could offer valuable data support for the population genetic structure of *T. absoluta*.

The SNP-transcripts are mainly involved in peroxisome, RNA transport, carbon metabolism, and protein processing in the endoplasmic reticulum. The results indicate that these SNP loci may be related to detoxification, energy metabolism, and self-repair functions. These SNP-transcripts increase resistance to pesticide susceptibility and protect *T. absoluta* from oxidative stress, accelerate the transport of RNA molecules, and help maintain normal energy metabolism and cell growth. These results could aid future research to determine the molecular mechanisms of *T. absoluta* response to pesticide susceptibility, promote the development of SNP molecular markers, and help construct genetic linkage maps.

## Figures and Tables

**Table 1:** Microsatellite distribution of full-length transcriptome *T. absoluta*.

Microsatellite or SSR Distribution	Data
Total number of sequences examined	60,386
Total size of examined sequences (bp)	164,477,748
Total number of identified SSRs	25,123
Occurrence frequency (%)	28.62
Appearance frequency (%)	41.6
Number of SSR containing sequences	17,284
Number of sequences containing more than 1 SSR	4824
Number of SSRs present in compound formation	2028

**Table 2:** The statistics of the type and number of SSR loci repetitions in *T. absoluta*.

Unit Size	Number of SSRs	Proportion (%)	Frequency (%)
mononucleotide	13,810	54.97	22.87
dinucleotide	4475	17.81	7.41
trinucleotide	4567	18.18	7.56
tetranucleotide	1944	7.74	3.22
pentanucleotide	178	0.71	0.29
hexanucleotide	149	0.59	0.25

## Data Availability

The raw data of the transcriptome sequence are openly available in NCBI SRA database (https://dataview.ncbi.nlm.nih.gov/object/PRJNA869533?reviewer=ikjih8ij3gupsg5ipnd3pgjtm4, accessed on 1 May 2022) at project number PRJNA869533. The data and materials supporting the conclusions of this study are included within the article.

## Supplementary Materials

The following supporting information can be downloaded at: https://www.mdpi.com/article/10.3390/insects16050446/s1, Figure S1: GO classification of SSR-transcripts in *T. absoluta*. Figure S2: COG pathway analysis of SSR-transcripts in *T. absoluta*. Figure S3: Secondary classification analysis of KEGG metabolic pathway of SSR-transcripts in *T. absoluta.* Figure S4: Comparison of the top four quantities of SNP-transcripts enrichment in GO annotations between the control group and each treatment group (a: Binding (molecular function); b: Catalytic activity (molecular function); c: Cellular process (biological process); d: Cetabotic process (biological process)). Figure S5: COG functional classification of SNP-transcripts in *T. absoluta* (A: Functional classification of the COG for SNP-transcripts in the CK; B: Functional classification of the COG for SNP-transcripts in the LC_10_; C: Functional classification of the COG for SNP-transcripts in the LC_30_; D: Functional classification of the COG for SNP-transcripts in the LC_50_). Figure S6: KEGG pathway analysis of SNP-transcripts for CK treatment of *T. absoluta.* Figure S7: KEGG pathway analysis of SNP-transcripts for the LC_30_ treatment of *T. absoluta*. Figure S8: KEGG pathway analysis of SNP-transcripts for the LC_50_ treatment of *T. absoluta*. Table S1: Frequency of identified SSR motifs in *T. absoluta*. Table S2: The six types of SSR repeat motifs and their frequencies in *T. absoluta*. Table S3: KEGG metabolic pathway classification of SSR-transcripts in *T. absoluta*. Table S4: Number of SNP loci in *T. absoluta*.
