# Supplementary figures and images for: The Characteristics and Functions of SSRs and SNPs Based on the Transcriptome of Tuta absoluta Exposed to Different Concentrations of Abamectin and Chlorantraniliprole
Source: Insects. 2025 Apr 24;16(5):446. doi: 10.3390/insects16050446 (PMC12112055; doi:10.3390/insects16050446)

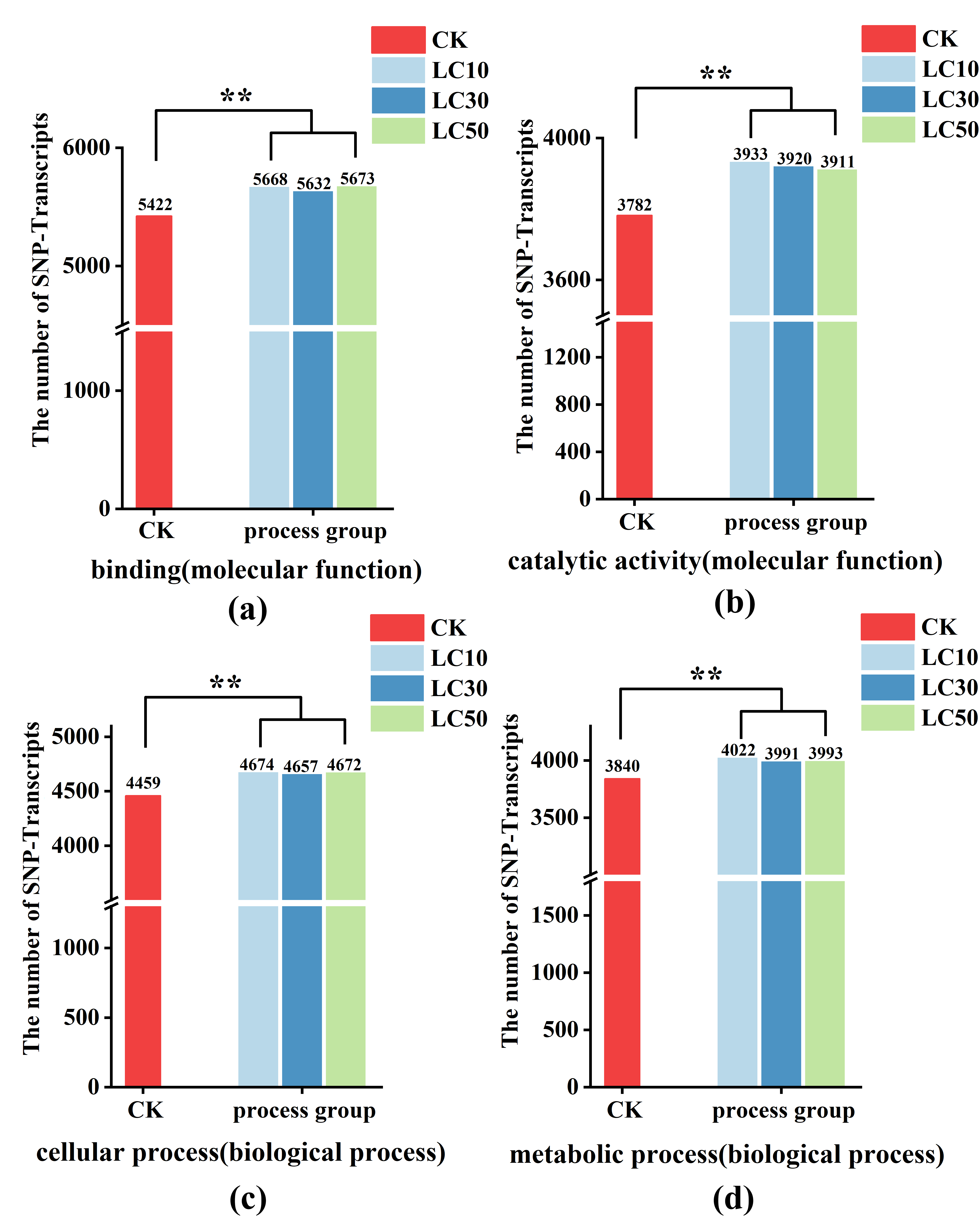

Supplement: Supplementary file 1 [file insects-16-00446-s001.zip › Figure S4 Comparison of the top four quantities of SNP-transcripts enrichment in GO annotations between the control group....tif]
